# Role of HMGB1/TLR4 Axis in Ischemia/Reperfusion-Impaired Extracellular Glutamate Clearance in Primary Astrocytes

**DOI:** 10.3390/cells9122585

**Published:** 2020-12-03

**Authors:** Chia-Ho Lin, Han-Yu Chen, Kai-Che Wei

**Affiliations:** 1 Master and PhD Programs in Pharmacology and Toxicology, School of Medicine, Tzu Chi University, Hualien 970, Taiwan; chlin97@mail.tcu.edu.tw (C.-H.L.); robinson.chen@integrated-bio.com (H.-Y.C.); 2 Department of Pharmacology, School of Medicine, Tzu Chi University, Hualien 970, Taiwan; 3 Department of Dermatology, Kaohsiung Veterans General Hospital, Kaohsiung 802, Taiwan; 4 School of Medicine, National Yang-Ming University, Taipei 112, Taiwan; 5 Faculty of Yuh-Ing Junior College of Health Care and Management, Kaohsiung 802, Taiwan

**Keywords:** astrocyte, glutamate reuptake, high-mobility group box 1, ischemic stroke, oxygen-glucose deprivation/reoxygenation, toll-like receptor 4

## Abstract

(1) Background: Abnormal accumulation of extracellular glutamate can occur as dysfunction of astrocytic glutamate transporters, which has been linked to ischemic brain injury. Excessive extracellular glutamate-induced abnormal excitotoxicity is the major cause of secondary neuronal damage after cerebral ischemia/reperfusion. However, the definite mechanism of impaired astrocytic glutamate reuptake remains unclear. (2) Methods: We investigated the mechanism of the HMGB1/TLR4 axis in extracellular glutamate clearance in primary astrocytes exposed to ischemia/reperfusion by using OGD/R (oxygen-glucose deprivation/reoxygenation) model. (3) Results: OGD/R insult activated the HMGB1/TLR4 axis for reducing the activity of glutamate clearance by inhibiting GLAST (glutamate aspartate transporter) expression in primary astrocytes. Interestingly, OGD/R-untreated astrocytes showed impairment of glutamate clearance after exposure to exogenous HMGB1 or conditioned medium from OGD/R-treated astrocytes culture. Inhibition of HMGB1 or TLR4 effectively prevented impaired glutamate clearance, which was induced by OGD/R, exogenous HMGB1, or conditioned medium from OGD/R-treated astrocytes. Furthermore, glycyrrhizic acid attenuated OGD/R-induced impairment of astrocytic glutamate clearance mediated by the HMGB1-TLR4 axis. (4) Conclusion: The HMGB1/TLR4 axis is a potential target for the treatment of post-ischemic excitotoxicity caused by GLAST dysfunction in astrocytes.

## 1. Introduction

Astrocytes are the largest population of glial cells in the central nervous system (CNS) and have several critical functions, including synthesis, secretion, re-uptake, and recycling of neurotransmitters. Under normal physiological conditions, neurotransmitters that are released in the synaptic cleft are immediately removed by astrocytes. This function is critical for ensuring effective synaptic process and the maintenance of neuronal excitability [1]. During ischemic stroke of the brain, abnormal neuronal excitability and excessive retained neurotransmitters exacerbate the severity of neural damage; in addition, ischemia-induced dysfunction of astrocytic reuptake of neurotransmitters is observed [2,3,4]. One of the important excitatory neurotransmitters is glutamate. Retained glutamate causes the overstimulation of neurons and is highly toxic for neurons, and excessive glutamate can induce apoptosis and death of neurons. Thus, astrocytes represent an important target for treating ischemic stroke via reducing excessive extracellular glutamate-induced neuronal damage such as cytotoxic edema [5].

The astrocytes uptake glutamate through specific glutamate transporters, namely glutamate transporter 1 (GLT-1) and glutamate aspartate transporter (GLAST) [6] in a glutamate-glutamine cycle [7,8]. During ischemic stroke of the brain, the decrease in oxygen and glucose levels induces glutamate levels to increase in the brain tissue, resulting in excitotoxic insult to the neurons. In the early phase of ischemic brain injury, the supply of oxygen and glucose stops, which results in a decrease in the ATP production of neuronal cells leading to inhibition of the Na+/K+-ATPase function. This inhibition causes severe loss of ionic gradients rendering to neuronal depolarization, which leads to excessive release of glutamate to induce excitotoxicity in neuronal cells by overstimulation of NMDA receptors [9]. Shortly following the insult, GLAST and GLT-1 are down-regulated for precipitating glutamate-mediated excitotoxic conditions [10,11]. In our previous study, we confirmed that oxygen-glucose deprivation/reoxygenation (OGD/R) induces dysfunction of astrocytic glutamate reuptake [12]; however, the exact mechanisms remain unclear.

Another puzzle is the mechanism of stroke-induced reperfusion injury and its profound damage to the surrounding nonischemic brain tissue. Inflammation, post-ischemic death signaling, and toxin release are the proposed mechanisms. However, anti-inflammatory agents and antioxidants provide limited effects in preventing the reperfusion injury. It is hypothesized that the abnormal astrocytic function and hyper-excitation of non-ischemic neurons play an important role, but the exact mechanism remains unclear.

We used OGD/R as an in vitro model to mimic scenarios of ischemia and reperfusion similar to that of in vivo condition. High mobility group box 1 (HMGB1), a cytokine-like mediator, exhibits pro-inflammatory activity and has been reported to be involved in ischemic brain neuronal damage and neuroinflammation [13]. However, its role in regulating the function of astrocytic glutamate clearance after ischemia/reperfusion injury remains unclear. In addition, there is evidence indicating that HMGB1-toll-like receptor 4 (TLR4) signaling participates in neuronal cell death and glutamate neurotoxicity [14]; however, its role in astrocytic dysfunction during ischemic stroke has not been reported before.

Using the in vitro model of OGD/R, we explored the mechanism of signaling pathways involved in the HMGB1-TLR4 activation of glutamate transporter and its correlation with reperfusion injury.

## 2. Materials and Methods

The animal study design was approved by the animal care committee of Tzu-Chi University (The approval number: 101082). The study was conducted by following the guide for the Care and Use of Laboratory Animals (National Institutes of Health Publications, No. 80-23) and the European Convention for the Protection of Vertebrate Animals used for Experimental and Other Scientific Purposes (ETS No. 123) for reducing animal suffering and the number of animals used.

### 2.1. Materials

Dulbecco’s modified eagle medium (DMEM)/Ham’s F12 medium supplemented with 2% fetal bovine serum (FBS) (Gibco BRL, Scotland, UK), tissue-culture dishes, and 96-well microtiter immunoplates (Nunc, Roskilde, Denmark) were purchased. Antibodies used in the study were for GLAST (1:2000, Chemicon, Temecula, CA, USA), β-actin (1:3000, Cell Signaling, Beverly, MA, USA), TLR4 (1:1000, Proteintech, Chicago, IL, USA), and HMGB1 (1:2000, Proteintech, Chicago, IL, USA). Eritoran and lipopolysaccharide from *Rhodobacter sphaeroides* (LPS-RS) were purchased from Calbiochem (San Diego, CA, USA). Recombinant HMGB1 (rHMGB1) was obtained from Sino Biological Inc. (Beijing, China).

Commercial kits, including HMGB1 ELISA (Elabscience Biotechnology Co., Shanghai, China), lactate dehydrogenase (LDH) release assay (Nanjing Jiancheng Bioengineering Institute, Nanjing, China), and Bio-Rad protein assay (Assay Kit II #5000002, Bio-Rad Laboratories, Feldkirchen, Germany), were used following the manufacturers’ instructions.

Immunoreactivities of HMGB1 siRNA and control siRNA (Santa Cruz Biotechnology, Inc., Santa Cruz, CA, USA) were detected using a Western blot chemiluminescence reagent system (Perkin-Elmer, Boston, MA, USA).

### 2.2. Culture of Primary Astrocytes

Primary astrocytes were isolated from the cerebral cortex of 3-day-old Sprague-Dawley neonate rat pups. The mixtures of cortical cells were initially suspended in a modified DMEM with 10% FBS, 30 mM glucose, 2 mM glutamine, and 1 mM pyruvate, and then plated on poly-D-lysine-coated T-75 tissue culture flasks at a density of 5 × 10^6^ cells/cm^2^. Classical astrocytes, forming a monolayer, were harvested 14–21 days after the plating, while the debris and other brain cells such as oligodendrocytes were removed from the flasks by shaking and changing the medium every 3–4 days [12].

Astrocytes were subsequently dissociated by trypsinization and were then plated on poly-l-lysine-coated 96-well micro-titer plates at a density of 2 × 10^5^ cells/cm^2^ (for glutamate clearance and LDH releasing test) and 60-mm culture dishes at a density of 1 × 10^6^ cells/cm^2^ (for siRNA transfection and immunoblot analysis). Approximately seven days after reseeding, astrocytes became confluent. The medium was then changed to modified DMEM containing 10% horse serum (gelding), followed by another seven days of incubation [12]. This method ensures that more than 98% of the cells in the culture are pure astrocytes identified by the presence of classic morphology (flattened and polygonal appearance) and the staining of glial fibrillary acidic protein, a specific astrocyte marker.

### 2.3. Oxygen-Glucose Deprivation/Reoxygenation

In the OGD/R group, the primary astrocytes were washed four times with glucose-free balanced salt solution (BSS) and incubated with the OGD solution, which was glucose-free BSS containing 116 mM NaCl, 5.4 mM KCl, 0.8 mM MgSO_4_, 1.0 mM NaH_2_PO_4_, 26.2 mM NaHCO_3_, 1.8 mM CaCl_2_, 0.01 mM glycine, and 10 mg/L phenol red, and were bubbled with N_2_/CO_2_ (95%/5%) for removing residual oxygen. The cells were placed in an airtight chamber (approximately 12 L in volume) with a continuous flux of anaerobic gas (95% N_2_/5% CO_2_) equilibrated for 10 min. The sealed chamber was then moved to an incubator at 37 °C for 6 h. After the chamber was opened, the cells were reoxygenated and put back to normal culture condition for 6–48 h for subsequent experiments. In contrast, the control primary astrocytes were incubated with BSS (containing 10 mM glucose), which was not bubbled with anaerobic gas.

### 2.4. Preparation of OGD/R Conditioned Medium

The primary astrocytes were treated with OGD for 6 h and then moved to normal culture condition (as reoxygenation) for 24 h. The conditioned medium (CM) was collected and centrifuged at 3500 rpm for 15 min at 4 °C to remove the debris, and then stored at −80 ℃ for the quantification of HMGB1 and treatment of astrocyte cultures.

### 2.5. LDH Release Assay

The supernatants of OGD/R-treated astrocytes were harvested for analyzing LDH release by measuring its absorbance at 450 nm using a microplate reader (BioTek Instrument, Winooski, VT, USA).

### 2.6. Assay for Astrocytic Glutamate Clearance

At 4 h after the addition of exogenous 200 µL of glutamate in the medium, the concentration of residual glutamate was determined using a colorimetric method modified by Lin et al. [7].

### 2.7. siRNA and Cell Transfection

The sequences of the control siRNA did not induce degradation of any known cellular mRNA. A pool of three target-specific 18–19 nucleotide siRNAs designed to knock down HMGB1 gene expression was used.

The sequences for HMGB1 siRNA (Santa Cruz Biotechnology) were designed as follows: sequence 1: 5′-GCAUAUUAGUACCAGUUGU-3′; sequence 2: 5′-CUGCUUAGUUUAGGGAACA-3′; sequence 3: 5′-GAGUCCUGGAUGAUACUAA-3′.

The sequence of TLR4 siRNA (Dharmacon Research, Lafayette, CO, USA) was 5′-ACGCUGUUCUGCUCAGGAGdTdT-3′. All siRNAs were prepared and used for cell cultures according to the manufacturers’ instructions. All experiments were performed at 36 h after siRNAs transfection, and the effect of the siRNAs on the target gene was verified using Western blotting.

### 2.8. Western Blot Assay

Primary astrocytes were lysed with a lysis buffer (50 mM Tris-HCl, pH 7.5, 150 mM NaCl, 1% Nonidet P-40, 0.5% deoxycholic acid, 0.1% SDS, 1 mM phenyl methyl sulfonyl fluoride, and 100 µg/mL leupeptin). The lysate was centrifuged at 19,720× *g* for 10 min. Supernatants were harvested, 40 microgram of protein was used for loading in Western blot, subjected to electrophoresis on SDS-polyacrylamide gel (8.5% for GLAST (60 kDa) and TLR4 (90 kDa); or 12% for HMGB1 (29 kDa)), and transferred to a nitrocellulose membrane. Non-fat dry milk solution (5%) was used as a blocking agent for 60 min; it was reacted with primary antibodies overnight at 4 °C and then incubated with HRP-conjugated secondary antibodies for 1 h at room temperature. Multiple exposures of films were used to ensure the optimum density, but not saturated density.

### 2.9. Statistical Analyses

Data were presented as mean ± standard error of the mean (SEM). Statistical significance was examined with an analysis of variance (one way ANOVA) followed by the Holm–Sidak test. The post hoc corrections by Mann–Whitney U tests based on the Bonferroni correction method for controlling overall type-I error were performed. A *p*-value of <0.05 was considered statistically significant.

## 3. Results

### 3.1. OGD/R Increased the Expression and Release of HMGB1 Protein and Decreased the Function of Glutamate Clearance in Primary Astrocytes

**Figure 1:**
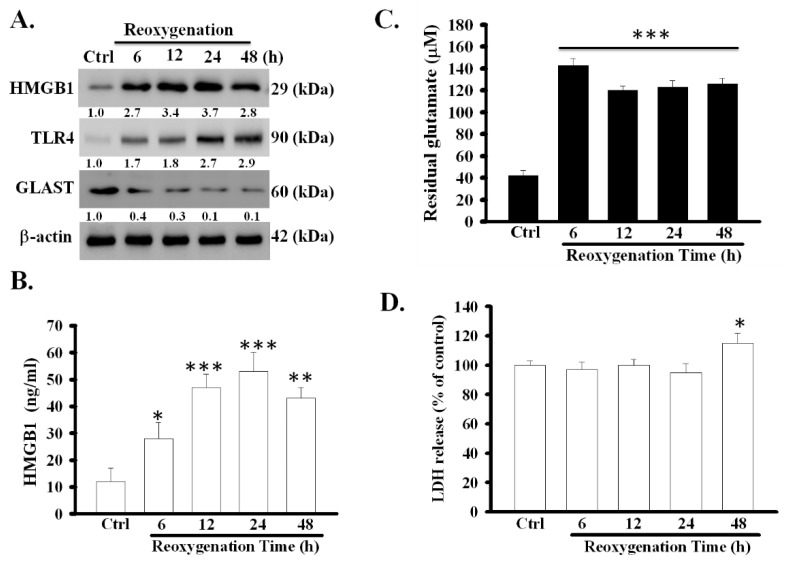
Effects of OGD/R on the expression of HMGB1 and TLR4 and the function of glutamate clearance in primary astrocytes. Primary astrocytes were exposed to OGD for 6 h followed by returning the cells to the normal cell culture condition (reoxygenation for 6–48 h). Thereafter, the expression of HMGB1, TLR4 and GLAST protein (**A**) were examined using Western blot. HMGB1 secretion (**B**) was analyzed using ELISA. The activity of glutamate clearance (**C**) was measured at each indicated time points after reoxygenation. Cell damage was assayed by measuring LDH release (**D**) * *p* < 0.05, ** *p* < 0.01, *** *p* < 0.001 vs. control group (ctrl). Data are presented as mean ± SEM, *n* = 6–8. OGD/R, oxygen-glucose deprivation/reoxygenation; HMGB1, high-mobility group box 1; TLR4, toll-like receptor 4; GLAST, glutamate aspartate transporter; LDH, lactate dehydrogenase; *n*, number of repeated examinations. The protein levels of HMGB1, TLR4 and GLAST were quantified by densitometric scanning of Western blots and normalized to β-actin in graph of Figure 1A.

The expression of HMGB1 in cell lysates and the levels of soluble HMGB1 in astrocytic medium significantly increased at various time points during reoxygenation (Figure 1A,B). It increased to a peak level at 24 h after reoxygenation (*p* < 0.001). Particularly, the increase was accompanied by a decrease in the activity of astrocytic glutamate clearance and the expression of GLAST protein (*p* < 0.001) (Figure 1A,C). To clarify whether the release of HMGB1 was derived from massive cell death, the degree of cell damage during reoxygenation was examined using the LDH release assay. LDH release during the first 6–24 h of reoxygenation did not alter; however, it slightly increased (*p* < 0.05) 48 h later (Figure 1D). These results revealed that OGD/R can simultaneously stimulate the active release of HMGB1 and inhibit the function of glutamate clearance in primary astrocytes, and only slightly result in cell damage at 48 h after reoxygenation.

### 3.2. HMGB1 Knockdown Prevented Dysfunction of Astrocytic Glutamate Clearance Induced by OGD/R

**Figure 2:**
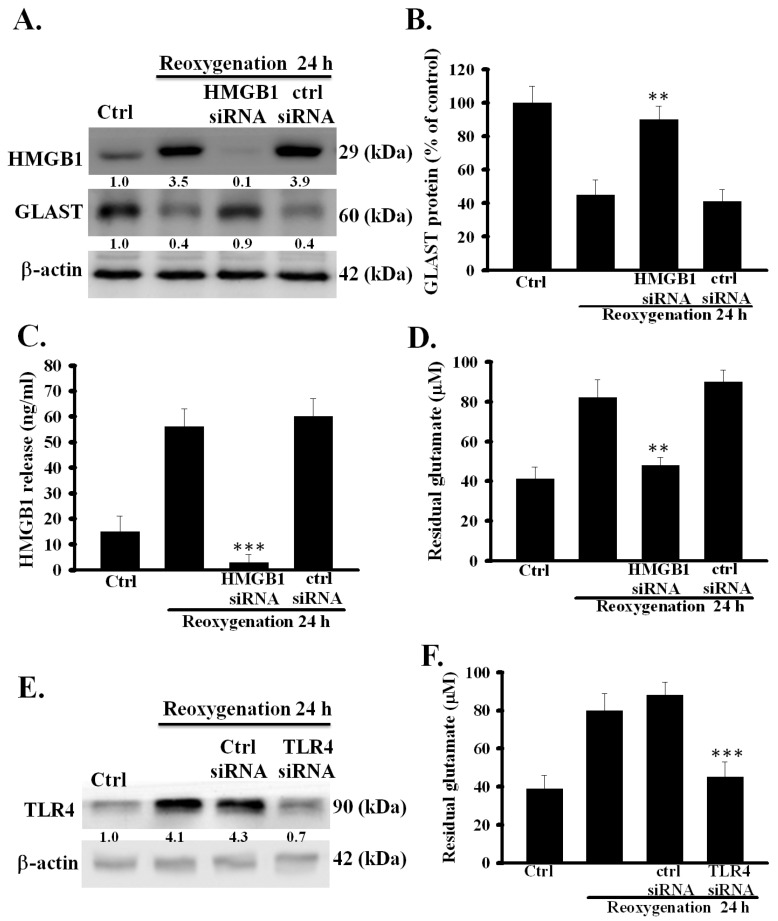
Effects of HMGB1 knockdown on OGD/R-impaired function of glutamate clearance in primary astrocytes. HMGB1 and control siRNA-treated astrocytes were exposed to OGD for 6 h followed by reoxygenation for 24 h. Thereafter, the expression of HMGB1 and GLAST protein (**A**) was examined using Western blot. Western blot of GLAST (**B**) was quantified using densitometry and ImageJ software. HMGB1 secretion (**C**) was analyzed using ELISA. The activity of glutamate clearance (**D**) was assayed at 24 h after reoxygenation. TLR4 and control siRNA-treated astrocytes were exposed to OGD/R insult. The expression of TLR4 (**E**) was examined by Western blot 24 h later. Activity of glutamate clearance in TLR4 and control siRNA-treated astrocytes was assayed at 24 h after reoxygenation (**F**). ** *p* < 0.01, *** *p* < 0.001 vs. control siRNA group. Data are presented as mean ± SEM, *n* = 6–8. OGD/R, oxygen-glucose deprivation/reoxygenation; HMGB1, high-mobility group box 1; TLR4, toll-like receptor 4; GLAST, glutamate aspartate transporter; *n*, number of repeated examinations.

To investigate the relationship between the upregulation of HMGB1 expression and the dysfunction of glutamate clearance induced by OGD/R in primary astrocytes, the effects of HMGB1 knockdown by siRNA on OGD/R-induced dysfunction of glutamate clearance were examined. First, the inhibitory effect of HMGB1 siRNA on the OGD/R-induced increase in the expression and release of HMGB1 was evaluated. As shown in Figure 2A,C, HMGB1 siRNA, but not control siRNA, strongly inhibited the up-regulation and secretion of HMGB1 in OGD/R-treated astrocytes (*p* < 0.001). Second, the effects of HMGB1 knockdown on the OGD/R-inhibited expression and activity of astrocytic glutamate transporters were examined. As shown in Figure 2A,B,D, the inhibition of HMGB1 expression significantly inhibited OGD/R-induced inhibition of GLAST expression and the activity of glutamate clearance (*p* < 0.001). These results suggest that the OGD/R-induced dysfunction of glutamate clearance was closely correlated with HMGB1 up-regulation in primary astrocytes.

### 3.3. Exogeneous Recombinant HMGB1 Inhibited the Activity of Glutamate Clearance in Primary Astrocytes

**Figure 3:**
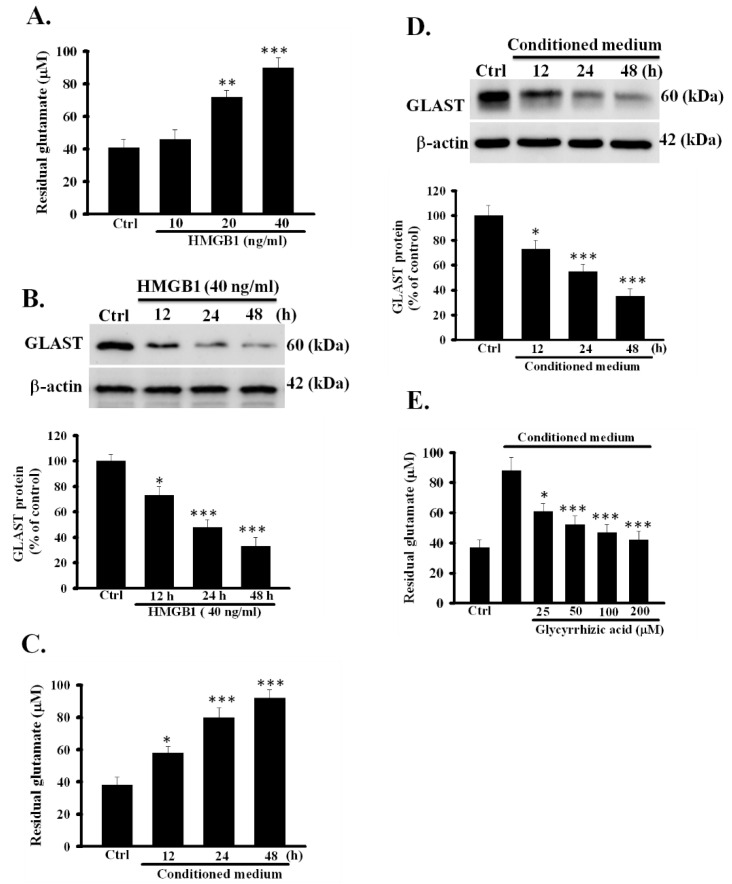
Effects of exogeneous recombinant HMGB1 and CM on function of glutamate clearance in primary astrocytes. (**A**) primary astrocytes were treated with rHMGB1 10–40 ng/mL, and the activity of glutamate clearance was assayed 24 h later. (**B**) primary astrocytes were incubated with 4 ng/mL rHMGB1 for 24 h, and GLAST protein was examined using Western blotting. (**C**) CM was collected from OGD/R-treated astrocytic culture and transferred to primary astrocytes to incubate for 12–48 h. Thereafter, the activity of glutamate clearance was assayed at each indicated time point. (**D**) Expression of GLAST protein in CM-treated astrocytes was assayed at each indicated time point by Western blotting. (**E**) Primary astrocytes were exposed to CM with or without glycyrrhizic acid (25–200 μM) and the activity of astrocytic glutamate clearance was assayed 24 h later. * *p* < 0.05, ** *p* < 0.01, *** *p* < 0.001 vs. control group (ctrl). Data are presented as mean ± SEM, *n* = 6–8. OGD/R, oxygen-glucose deprivation/reoxygenation; rHMGB1, recombinant high-mobility group box 1; GLAST, glutamate aspartate transporter; CM, conditioned medium; *n*, number of repeated examination.

To further verify the inhibitory effect of OGD/R-released HMGB1 on the function of astrocytic glutamate clearance, exogenous rHMGB1 was investigated as to whether it alone could inhibit the activity of astrocytic glutamate clearance. rHMGB1 concentration-dependently reduced the activity of astrocytic glutamate clearance significantly (*p* < 0.001) (Figure 3A). A previous study showed that GLAST protein is the major glutamate transporter, and it is responsible for the removal of most extracellular glutamate in primary astrocytes [12]. Thus, the effect of rHMGB1 on GLAST protein expression was examined in our study. As shown in Figure 3B, rHMGB1 remarkably and time-dependently inhibited GLAST protein expression (*p* < 0.001). These results further confirmed that HMGB1 could reduce the activity of glutamate clearance by inhibiting GLAST protein expression in primary astrocytes. 

### 3.4. Conditioned Medium Inhibited the Function of Astrocytic Glutamate Clearance 

To demonstrate that HMGB1 released by OGD/R can also inhibit glutamate clearance capacity in non OGD-exposed normal astrocytes, the effect of CM from the culture of OGD/R-treated astrocytes on the function of glutamate clearance in normal astrocytes was examined. As shown in Figure 3C, the activity of glutamate clearance in CM-treated astrocytes was significantly and time-dependently inhibited (*p* < 0.001). In addition, the expression levels of GLAST protein were significantly reduced in CM-treated astrocytes (Figure 3D, *p* < 0.001), suggesting that the function of astrocytic glutamate clearance can indeed be inhibited by OGD/R-treated CM.

Glycyrrhizic acid, a well-known HMGB1 inhibitor, can bind directly to HMGB1 and inhibit its biological activity [15,16]. Hence, the inhibition of HMGB1 activity by glycyrrhizic acid was investigated for if it could inhibit the CM-induced dysfunction of astrocytic glutamate clearance. As shown in Figure 3E, glycyrrhizic acid could effectively and concentration-dependently inhibit the CM-impaired activity of astrocytic glutamate clearance (*p* < 0.001). These results further confirmed that HMGB1 is indeed an important mediator in OGD/R-induced dysfunction of glutamate clearance in primary astrocytes.

### 3.5. TLR4 Was Involved in OGD/R-Induced Dysfunction of Astrocytic Glutamate Clearance

HMGB1-TLR4 signaling pathway has been reported to play an important role in mediating OGD/R-evoked astrocytic swelling and cytokine release [17]. Whether the signaling pathway participates in regulating the function of astrocytic glutamate clearance has not yet been investigated. Hence, the role of TLR4 in the OGD/R-induced dysfunction of glutamate clearance was examined in primary astrocytes. The effect of OGD/R on astrocytic TLR4 expression is shown in Figure 1A. OGD/R significantly and time-dependently increased TLR4 levels at several measured time points after reoxygenation. In addition, the efficacy of siRNA for suppressing TLR4 expression in OGD/R-treated primary astrocytes was tested. TLR4 siRNA, but not control siRNA, effectively suppressed OGD/R-increased astrocytic TLR4 expression (Figure 2E). Next, the effects of TLR4 suppression by siRNA on the OGD/R-inhibited activity of astrocytic glutamate clearance was examined. Figure 2F shows that TLR4 suppression significantly prevented the OGD/R-induced inhibition of the activity of glutamate clearance (*p* < 0.001). These results suggest that TLR4 was also involved in the OGD/R-induced dysfunction of glutamate clearance in primary astrocytes.

### 3.6. Inhibition of TLR4 Prevented rHMGB1-Induced Inhibition of the Function of Astrocytic Glutamate Clearance 

**Figure 4:**
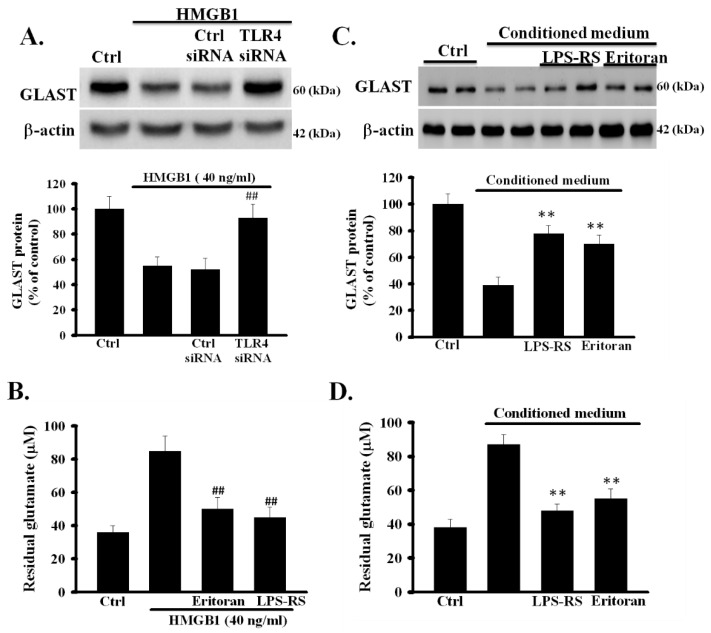
Role of TLR4 on HMGB1 and CM-impaired function of glutamate clearance in primary astrocytes. (**A**) Effects of TLR4 knockdown on rHMGB1-reduced expression of GLAST. TLR4 and control siRNA-treated astrocytes were exposed to rHMGB1 (40 ng/mL) for 24 h and the expression levels of GLAST were examined using Western blot. (**B**) Effects of TLR4 inhibitors on rHMGB1-inhibited activity of astrocytic glutamate clearance. Primary astrocytes were exposed to rHMGB1 (40 ng/mL) with or without TLR4 inhibitors, eritoran or LPS-RS, and the activity of glutamate clearance was assayed 24 h later. (**C**) Primary astrocytes were exposed to CM with or without TLR4 inhibitors, eritoran or LPS-RS, for 24 h. Thereafter, the expression levels of GLAST protein were examined using Western blot. (**D**) Activity of astrocytic glutamate clearance was assayed by measuring the residual glutamate in CM. ^##^ *p* < 0.01 vs. rHMGB1 alone, ** *p* < 0.01 vs. CM alone. Data are presented as mean ± SEM, *n* = 6. OGD/R, oxygen-glucose deprivation/reoxygenation; HMGB1, high-mobility group box 1; rHMGB1, recombinant HMGB1; CM, conditioned medium; TLR4, toll-like receptor 4; GLAST, glutamate aspartate transporter; *n*, number of repeated examinations; LPS-RS, lipopolysaccharide from *Rhodobacter sphaeroides.*

To investigate whether TLR4 is involved in rHMGB1-reduced expression of GLAST, TLR4 siRNA- and control siRNA-treated astrocytes were exposed to rHMGB1 (40 ng/mL) for 24 h and the levels of GLAST protein were analyzed using Western blot. As shown in Figure 4A, the levels of GLAST in control siRNA-treated astrocytes, but not in TLR4 siRNA-treated astrocytes, were significantly reduced (*p* < 0.001). To further clarify whether the activation of TLR4 is required for the exogenous rHMGB1-induced inhibition of the activity of astrocytic glutamate clearance, the effects of TLR4 inhibitors on rHMGB1-inhibited activity of glutamate clearance were examined in primary astrocytes. Primary astrocytes were treated with rHMGB1 (40 ng/mL) in the presence or absence of TLR4 inhibitors (eritoran and LPS-SR, both 1 µg/mL), and the activity of glutamate clearance was examined 24 h later. As shown in Figure 4B, TLR4 inhibitors effectively inhibited the rHMGB1-induced inhibition of the activity of astrocytic glutamate clearance (*p* < 0.001). These results revealed that the activation of the HMGB1-TLR4 signaling pathway could negatively regulate the function of glutamate clearance in cortical astrocytes.

### 3.7. Inhibition of TLR4 Prevented CM-Induced Inhibition of the Activity of Astrocytic Glutamate Clearance

We further investigated whether TLR4 was involved in CM-induced dysfunction of astrocytic glutamate clearance. Cortical astrocytes were treated with CM with or without TLR4 inhibitors, eritoran and LPS-SR, and the function of glutamate clearance was examined 24 h later. As shown in Figure 4C, the CM-reduced expression of GLAST was inhibited by TLR4 inhibitors (*p* < 0.01). In addition, TLR4 inhibitors significantly prevented the CM-induced inhibition of the activity of astrocytic glutamate clearance (*p* < 0.01) (Figure 4D). These results indicated that OGD/R-released HMGB1 could directly affect the function of glutamate clearance of normal astrocytes by activating TLR4.

## 4. Discussion

In our study, the OGD/R of astrocytes induced impairment (dysfunction) in astrocytic glutamate clearance, which was mediated by the HMGB1-TLR4 signaling axis. Moreover, it caused astrocytes to secrete damaged factor HMGB1 that affected healthy astrocytes to show impaired astrocytic glutamate clearance, which was also mediated via the HMGB1-TLR4 pathway.

HMGB1 is a damaged factor, which is actively secreted from ischemia/reperfusion -injured and stressed astrocytes. It promotes OGD/R responses by binding to key pattern TLR4. It is highly conserved among various species and consists of a single 215-amino acid polypeptide chain organized into two DNA-binding domains linked by a short basic hinge and an acidic C-terminal tail [18]. Two DNA-binding domains, L-shaped A-box and B-box, individually have unique inflammatory, regulatory, and cytokine stimulating functions. Surface plasmon resonance studies have indicated that the specific binding of HMGB1 to TLR4 is dependent on the cysteine 106 within the B-box [19,20]. These data suggest the B-box of HMGB1 protein to be the functional domain that interacts with TLR4; moreover, cysteine residue 106 within HMGB1 regulates its receptor-binding ability of OGD/R stress responses in ischemia injury.

OGD/R is a widely used cell model for mimicking the condition of cell death observed in a hypoxia-induced brain injury model, including ischemic stroke. We demonstrated that OGD/R astrocytes induced increased HMBG1-TLR4 signaling expression, while exogenous HMGB1 increased the dysfunction of glutamate clearance in OGD/R astrocytes. A critical role of HMGB1 in myocardial ischemia model is to induce tissue regeneration [21,22]. HMGB1 can exert a dual effect after ischemic injury by amplifying the inflammatory response, leading to tissue damage in the acute phase and by participating in tissue remodeling, leading to tissue repair in the late phase [23]. Furthermore, the application of neutralizing monoclonal antibodies against HMGB1 can attenuate the infarct size and severity after middle cerebral artery occlusion in rats [24,25]. The post-ischemic administration of a neutralizing antibody against HMGB1 protects against ischemic brain injury [26,27]. These investigations indicate that HMGB1 modulation may be a therapeutic target for brain ischemia and post-ischemic inflammation.

An in vitro model of OGD/reperfusion has been used for studying the mechanisms of neuron death after ischemic stroke. NMDA (*N*-methyl-d-aspartic acid) mediated excitotoxicity, and oxidative stress were reported to be involved in neuron death [28,29,30]. However, the roles of astrocytes in ischemic brain injury-induced excitotoxicity have been seldom investigated before. Our results showed that OGD/reoxygenation insult result in the inhibition of astrocytic glutamate clearance by activating HMGB1/TLR4 pathway to down-regulate expression of GLAST. These findings imply that activation of astrocytic HMGB1/TLR4 signaling may indirectly involve in excitotoxicity after ischemic stroke.

HMGB1 is an important mediator during and after ischemic stroke. In the early phase of ischemic stroke, neurons die, and subsequently, HMGB1 is released from the neurons for accelerating the inflammatory response. In addition, HMGB1 is modified to the restored or oxidized forms, which have specific cellular functions in the ischemic condition [24]. Umahara et al. found that HMGB1 was located in the neuronal cytoplasm in the acute stage of cerebral infarction in patients, while HMGB1 is mainly secreted by macrophages located in some ischemic regions in the late stage of cerebral infarction [31]. Previous studies have shown that hypoxia stimulation resulted in a change in the astrocytic transcriptome, which can modify mitochondria function and immune response [32,33]. These studies are similar to our results, in which OGD/R insult can activate HMGB1/TLR4 signaling to alter the function of glutamate clearance in primary astrocytes. Thus, hypoxia injury can directly affect the physiological function of astrocytes and cause neuronal damage. We report here that OGD/R-released HMGB1 inhibits the function of glutamate clearance in OGD/R astrocytes. This finding is consistent with that of a previous study in which HMGB1-TLR4 signaling contributed to neuronal cell death and glutamate neurotoxicity [14]. HMGB1 inhibited mouse neural glial glutamate transporters by GLAST neural activation particles [24]. All these results supported the hypothesis that the HMGB1 release from various immune cells, including astrocytes, is critical for affecting the nerves regeneration of the brain–immune interaction and causing glutamate-induced neurotoxicity after ischemia.

The pathological process of cerebral I/R injury is complex, and no effective treatments are available yet. Glycyrrhizic acid, an inhibitor of HMGB1, can directly bind HMGB1 without affecting other chemokines [34]. Our results indicate that glycyrrhizic acid showed a potentially protective effect on OGD/R astrocytes by inhibiting the secreted HMGB1 in the conditioned medium of OGD/R-treated astrocytes. Because HMGB1 impaired the reuptake and clearance of glutamate in astrocytes in response to ischemic injury, we suggest that glycyrrhizic acid can antagonize the impaired function and possibly act as a promising adjuvant therapy for stroke in the future.

There are a few limitations to this study. This was not a clinical study in humans, nor was it an in vivo experiment. This study is an in vitro study using primary astrocytes, which mimic the reperfusion injury following ischemic/reperfusion insults to the brain. In the future, further studies in animals are warranted. However, it is very difficult to dissect each type of cell into different scenarios in animals. Alternatively, we will adopt new innovative tools, such as a humanized 3D self-organized model and organoids/organ on a chip platform to especially enable advanced optical imaging [35,36], for the next step.

In conclusion, our research indicated that the HMBG1-TLR4 signaling mediated the OGD/R-induced impairment of astrocytic glutamate clearance. Targeting HMGB1 and its key pattern TLR4 may offer effective approaches for preventing neuronal deaths owing to cerebral I/R injury.

## Abbreviations

CM	conditioned medium;
GLAST	glutamate aspartate transporter;
GLT-1	glutamate transporter 1;
HMGB1	high-mobility group box 1;
OGD/R	oxygen-glucose deprivation /reoxygenation;
TLR4	toll-like receptor 4; LDH, lactate dehydrogenase;
DMEM	Dulbecco’s modified eagle medium;
FBS	fetal bovine serum;
BSS	balanced salt solution;
CM	conditioned medium;
SEM	standard error of the mean;
LPS-RS	lipopolysaccharide from *Rhodobacter sphaeroides*
